# Indole-3-Propionic Acid, a Gut-Derived Tryptophan Metabolite, Associates with Hepatic Fibrosis

**DOI:** 10.3390/nu13103509

**Published:** 2021-10-05

**Authors:** Ratika Sehgal, Mariana Ilha, Maija Vaittinen, Dorota Kaminska, Ville Männistö, Vesa Kärjä, Marjo Tuomainen, Kati Hanhineva, Stefano Romeo, Päivi Pajukanta, Jussi Pihlajamäki, Vanessa D. de Mello

**Affiliations:** 1 Department of Clinical Nutrition, Institute of Public Health and Clinical Nutrition, University of Eastern Finland, 70211 Kuopio, Finland; ratika.sehgal@uef.fi (R.S.); mariana.ilha@uef.fi (M.I.); maija.vaittinen@uef.fi (M.V.); dorota.kaminska@uef.fi (D.K.); marjo.tuomainen@uef.fi (M.T.); kati.hanhineva@utu.fi (K.H.); jussi.pihlajamaki@uef.fi (J.P.); 2 Departments of Medicine, University of Eastern Finland and Kuopio University Hospital, 70211 Kuopio, Finland; ville.mannisto@kuh.fi; 3 Department of Pathology, University of Eastern Finland and Kuopio University Hospital, 70211 Kuopio, Finland; vesa.karja@kuh.fi; 4 Department of Life Technologies, Food Chemistry and Food Development Unit, University of Turku, 20500 Turku, Finland; 5 Department of Molecular and Clinical Medicine, University of Gothenburg, 40530 Gothenburg, Sweden; stefano.romeo@wlab.gu.se; 6 Department of Human Genetics, David Geffen School of Medicine, University of California Los Angeles (UCLA), Los Angeles, CA 90095, USA; ppajukanta@mednet.ucla.edu; 7 Institute for Precision Health, School of Medicine, University of California Los Angeles (UCLA), Los Angeles, CA 90095, USA; 8 Department of Medicine, Endocrinology and Clinical Nutrition, Kuopio University Hospital, 70211 Kuopio, Finland

**Keywords:** indole-3-propionic acid, non-alcoholic fatty liver disease, hepatic fibrosis, hepatic stellate cells, gut microbiota

## Abstract

Background and Aims: Gut microbiota-derived metabolites play a vital role in maintenance of human health and progression of disorders, including obesity and type 2 diabetes (T2D). Indole-3-propionic acid (IPA), a gut-derived tryptophan metabolite, has been recently shown to be lower in individuals with obesity and T2D. IPA’s beneficial effect on liver health has been also explored in rodent and cell models. In this study, we investigated the association of IPA with human liver histology and transcriptomics, and the potential of IPA to reduce hepatic stellate cell activation in vitro. Methods: A total of 233 subjects (72% women; age 48.3 ± 9.3 years; BMI 43.1 ± 5.4 kg/m^2^) undergoing bariatric surgery with detailed liver histology were included. Circulating IPA levels were measured using LC-MS and liver transcriptomics with total RNA-sequencing. LX-2 cells were used to study hepatoprotective effect of IPA in cells activated by TGF-β1. Results: Circulating IPA levels were found to be lower in individuals with liver fibrosis compared to those without fibrosis (*p* = 0.039 for all participants; *p* = 0.013 for 153 individuals without T2D). Accordingly, levels of circulating IPA associated with expression of 278 liver transcripts (*p* < 0.01) that were enriched for the genes regulating hepatic stellate cells (HSCs) activation and hepatic fibrosis signaling. Our results suggest that IPA may have hepatoprotective potential because it is able to reduce cell adhesion, cell migration and mRNA gene expression of classical markers of HSCs activation in LX-2 cells (all *p* < 0.05). Conclusion: The association of circulating IPA with liver fibrosis and the ability of IPA to reduce activation of LX-2 cells suggests that IPA may have a therapeutic potential. Further molecular studies are needed to investigate the mechanisms how IPA can ameliorate hepatic fibrosis.

## 1. Introduction

The liver is a central hub for a multitude of metabolic functions that are critical for physiological homeostasis [1]. Hepatic lipid and glucose metabolism dysregulation, hallmarks of non-alcoholic fatty liver disease (NAFLD) sets in an inflammatory milieu, leading to severe tissue injury, remodeling, and fibrosis, as commonly seen in non-alcoholic steatohepatitis (NASH) [2,3,4].

The gut microbiota is critical for human health maintenance by regulating metabolic processes such as the degradation of dietary elements and production of microbial metabolites that form an integral part of the systemic metabolome [5,6]. Indole-3-propionic acid (IPA) is one of the gut-derived tryptophan metabolites associated with dietary fiber intake [7,8] that is found to be reduced in various metabolic diseases in humans [9,10,11,12,13]. Recent studies in humans and rodents conclude that IPA levels are potentially modulated by dietary fat and fiber intake and suggests improving IPA levels with dietary and lifestyle modifications [7,13,14,15]. IPA is also known to induce anti-oxidative, anti-inflammatory, anti-hyperglycemic, and neuro-protective effects in vitro and in vivo [16,17,18,19]. The hepatoprotective effects of IPA have been shown in few in vitro models using the renal proximal tubular cells and rat hepatic microsomal membranes [20,21]. Recent rodent studies highlight the beneficial role of IPA in ameliorating the advanced liver pathology including NASH and hepatocellular carcinoma (HCC) by altering the fibrotic and pro-inflammatory processes [15,22].

The gut-liver axis also plays an integral part in the progression of hepatic fibrosis and precipitation of other hepatic alterations [23,24,25]. Systemic circulating intestinal metabolites such as short-chain fatty acids, branched-chain amino acids, secondary bile acids, betaine, tryptophan and its metabolites, have been reported to be associated with NASH and HCC [26,27,28,29,30]. Despite this strong evidence, little is known about their impact on histological and molecular mechanisms contributing to NAFLD.

Liver fibrosis is a result of a dysregulated healing response and the primary cellular source of extracellular matrix (ECM) components are the hepatic stellate cells (HSCs), interacting actively with major cytokines [31,32]. Apoptosis of the activated HSCs or reversal to a quiescent state are critical steps in the reversal of fibrosis [31,33]. In the present study, we identified circulating IPA levels to be associated with liver inflammation and fibrosis in obese individuals. Thus, we investigated the association of circulating IPA with liver transcriptomics identifying potential link with pathways regulating extracellular matrix and fibrosis. Thereafter, we used an in vitro cell model of spontaneously immortalized human hepatic stellate cell line (LX-2) to reveal the potential of IPA to ameliorate activation of LX-2 cells and development of fibrosis in human NAFLD.

## 2. Materials and Methods

### 2.1. Study Population

The study population consisted of participants from the ongoing Kuopio Obesity Surgery (KOBS) Study [34]. A total of 233 subjects (mean ± SD: 48.3 ± 9.3 years old; body mass index, BMI: 43.1 ± 5.4 kg/m^2^; 64 males; 80 with T2D) undergoing bariatric surgery were included. Before the surgery, study subjects participated in a one-day visit and were interviewed for disease history and current drug treatments. Fasting blood samples were drawn after 12 h fasting and glucose, insulin and serum lipids were determined as described previously [34]. Liver biopsies were obtained during the bariatric surgery. Written informed consent was obtained from all participants and the study protocol was approved by the Ethics Committee of the Northern Savo Hospital District (54/2005, 104/2008, and 27/2010) and were in accordance with the Helsinki Declaration.

### 2.2. Liver Histology

Liver biopsies were obtained using Trucut needles (Radiplast AB, Uppsala, Sweden) or as a wedge biopsy during elective gastric bypass operations. Liver biopsies (*n* = 233) were scored for histology by an experienced pathologist according to the standard criteria, as described previously [35,36]. Steatosis and lobular inflammation were scored on a four-point scale (0,1,2, and 3), ballooning scored on a three-point scale (0,1, and 2) and hepatic fibrosis was scored on a five-point scale (0,1,2,3, and 4) [35]. To test the associations of circulating IPA levels with each of the liver histology (steatosis, lobular inflammation, ballooning, or fibrosis), we categorized each of them into two groups based on the presence (all except 0) or absence (only 0) of the respective stages/grades. Out of 233, 164 subjects could be categorized into distinct histological phenotypes: (1) Normal liver without any steatosis, inflammation, ballooning, or fibrosis (*n* = 79); (2) Simple steatosis (steatosis > 5%) without evidence of hepatocellular ballooning, inflammation or fibrosis (*n* = 40); (3) NASH (*n* = 45) as described previously [37] and summarized in Table 1.

### 2.3. Measurement of Serum Indole-3-Propionic Acid (IPA)

Fasting serum samples obtained at the baseline (before the surgery) were submitted to non-targeted LC-MS for metabolomics profiling (*n* = 233). The samples were analyzed with a UHPLC-qTOF-MS system (1290 LC, 6540 qTOF-MS, Agilent Technologies, Waldbronn, Karlsruhe, Germany) as described previously [37]. IPA was identified based on retention time and MS/MS spectral comparison with pure standard compound. To validate the approach of using IPA signal intensity, reported as peak area for our analysis we analyzed fasting serum IPA concentrations measurements in a sub cohort of 121 participants (described in Table S1) using a similar protocol as previously applied [38]. IPA signal intensity and concentration measurements correlated strongly (*r* = 0.82, *p* < 0.0001; Figure S1), and thus the IPA signal intensities (peak area) were considered for all further analysis.

### 2.4. Gene Expression in Liver Using RNA-Sequencing

Total RNA sequencing was performed for 175 liver samples from the current study population. Briefly, RNA sequencing libraries underwent 50-nucleotide long paired-end sequencing on Illumina HiSeq 2500 machine, followed by read alignment, normalization and differential expression analysis considering the technical and confounding factors (namely RIN, uniquely aligned reads %, 3’ bias, age, sex and BMI) as described previously [37,39].

### 2.5. Validation of Anti-Fibrotic Effects of IPA Using Human Hepatic Stellate Cells (LX-2)

The reagents and chemicals used for the experiments are listed in Supplementary Material (Methods S1). Immortalized human hepatic stellate cells (LX-2) were maintained in DMEM-1% Pen/Strep supplemented with 2% FBS and incubated at 37 °C in a humidified atmosphere supplied with 5% CO_2_.

#### 2.5.1. Cytotoxicity Assay

First, to assess the effect on LX-2 cells viability and cytotoxicity and select the working doses of IPA, 3-(4,5-dimethylthiazol-2-yl)-2,5-diphenyl tetrazolium bromide (MTT) assay was performed as per manufacturer’s protocol. Briefly, cells in 96-well plates (15,000 cells/well/100 μL) after overnight adhesion were treated with various doses of IPA (1 nM, 10 nM, 100 nM, 1 μM, 10 μM, 100 μM, 1 mM, 2 mM) and corresponding vehicle controls (DMSO). After 24 and 48 h of IPA treatment, media from each well was replaced with 100 μL of MTT (0.2 mg/mL), followed by two hours incubation. This was followed by replacement of MTT with DMSO to extract the formazan crystals. The absorbance was read using Cytation 3 (Biotek Instruments, USA) at 570 nm, reference background at 650 nm and using DMSO as control. After 24 h of treatment, only the highest dose of IPA (2 mM) significantly reduced cell viability. Whereas, after 48 h, both 1 and 2 mM reduced cell viability significantly. Therefore, IPA doses of 1, 10, and 100 μM were selected for the actual experiments, which were the highest non-toxic concentrations, close to the physiological range [19,38] and which have been previously used [15,40,41].

#### 2.5.2. Cell Adhesion Assay

To study the effect of IPA on cell adhesion, LX-2 cells were plated in 6-well plates (150,000 cells/well). Next day, the cells were treated with IPA and corresponding vehicle controls (DMSO). After 24 and 48 h of treatment, the cells were trypsinized and transferred to a 96-well plate. After two hours of adhesion, the media in each well was replaced with 100 µL of MTT (0.2 mg/mL), and the same protocol as mentioned above was followed.

#### 2.5.3. Wound Healing Cell Migration Assay

For cell migration, LX-2 cells were plated in 24-well plate (40,000 cells/well). After 24 h of plating, a scratch was made using the 200 µL pipette tip followed by DPBS wash and treatment with IPA and corresponding vehicle controls for 24 h. The wound was imaged at 0 and 24 h in Zeiss inverted light microscope (Zeiss Axio Vert.A1 and AxioCam MRm, Jena, Germany) at 50× magnification and area of the wounds were quantified using ImageJ software (National Institute of Health, Bethesda, MD, USA, Version 1.51).

#### 2.5.4. Activation of LX-2 Cells by TGF-β1 and IPA Treatment

For the induction of fibrogenesis, we incubated the LX-2 with a potent cytokine TGF-β1 as studied previously [42]. Briefly, the cells were plated in 24-well plate (40,000 cells/well) and were treated next day with TGF-β1 (5 ng/mL) in serum free media for 24 and 48 h and 4 nM HCL with 0.1% BSA was used as vehicle control for TGF-β1. The treatment to the cells with IPA (100 µM) for 24 h was performed either as a co-treatment with TGF-β1 induction or as a recovery after 24 h of TGF-β1 induction without media replacement.

#### 2.5.5. Quantitative RT-PCR

The activated and IPA treated cells along with the controls were lysed using RLT supplemented with 1% β-mercaptoethanol and total RNA was extracted according to the manufacturer’s instructions. RNA was then transcribed into complementary DNA (cDNA) and quantified for gene expression levels of α-smooth muscle actin (*αSMA*), collagen type 1 *(COL1A2)*, matrix metalloproteinase-2 *(MMP2)*, tissue inhibitor of metalloproteinases 1 *(TIMP1)* and integrin subunit alpha 3 *(ITGA3)* using specific forward and reverse primers listed in Table S2. Human 60S acidic ribosomal protein P0 (*RPLP0)* mRNA levels served as an internal control. The QuantStudio (QuantStudio 6 pro Real-Time PCR System, Thermo Fisher, Landsmeer, The Netherland) comparative CT (ΔΔCT) cycling parameters and 2^−∆∆Ct^ method was used to calculate relative fold gene expression.

### 2.6. Statistical Analysis

IPA signal intensities were inverse-rank normalized prior to the analysis. The associations of fasting serum IPA levels between stages/grades for each liver histology (steatosis, lobular inflammation, ballooning, fibrosis) were tested using general linear model (univariate). Certain models were further adjusted for age, gender, and BMI. All the analyses were performed using SPSS version 27 program (IBM Inc., Armonk, NY, USA) and two-sided *p* value of <0.05 was considered statistically significant.

To evaluate associations between global gene expression and clinical features, edgeR’s negative binomial generalized linear model with quasi-likelihood F test, controlling for technical and biological factors influencing gene expression identified with PCA was used. For the differential gene expression model, IPA signal intensities were used, as explained in 2.3. The following covariates were included in the analysis: uniquely aligned reads %, 3’ bias, and RNA integrity number (RIN), age, gender, and BMI. To reveal the functional relationship among key genes (*p* value < 0.01), two different approaches were employed. First, Ingenuity Pathway Analysis’ core analysis was used to find the top canonical pathways. Next, we used g:Profiler to extract the gene ontology (GO) and Reactome terms and assembled all the significantly enriched terms into functionally interpretable clusters using Enrichment Map (v3.1.2) plugin in Cytoscape (v3.7.1) [43].

For LX-2 experiments, data were obtained at least from three independent experiments done in triplicate and expressed as mean ± SD. Samples were tested for normality using D’Agostino and Pearson omnibus normality test. For each treatment group, corresponding controls were also included and analyzed, however for the simplicity of representation single control was used. One-way ANOVA followed by Bonferroni’s post hoc test was used for statistical comparisons using GraphPad Prism v.5 (GraphPad Software Inc., version 5, San Diego, CA, USA).

## 3. Results

### 3.1. Circulating IPA Levels Associate with the Lobular Inflammation and Fibrosis

The main characteristics of the KOBS study population divided in those with normal liver, simple steatosis (SS) and NASH liver phenotypes are shown in Table 1 (*n* = 164). IPA levels were not significantly different among the three liver phenotypes, as published in our previous publication in this cohort [37].

**Figure 1:**
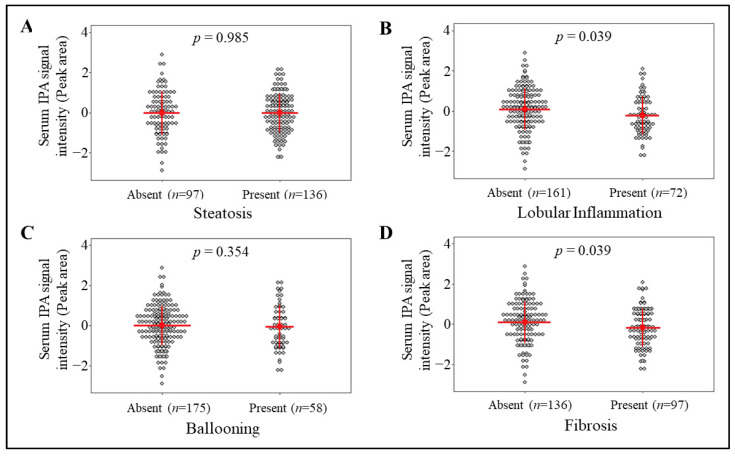
Associations of serum IPA signal intensity with liver histology, based on presence (all except 0) or absence (stage/grade 0) of each specific histological characteristic. Dot plot presents the inverse-normalized serum IPA signal intensities (*y*-axis) across the (**A**) steatosis, (**B**) lobular inflammation, (**C**) ballooning and (**D**) fibrosis. Red lines and whiskers represent mean serum IPA signal intensity and SD, respectively. The number of individuals in each group are indicated for each group in parentheses. General linear model (univariate) test results *p* value is shown as *p* for each comparison between presence or absence of each specific histological characteristic.

The limitation of using these categories (Table 1) is that the association between IPA and specific histological findings defining NASH may be lost. Hence, to study associations of IPA specifically with presence or absence of steatosis, lobular inflammation, ballooning, and fibrosis was performed. In this analysis, all the participants with characterized liver histology (*n* = 233, Table S1) were included to assess the association of IPA signal intensities with detailed liver histology, based on presence (all except 0) or absence (stage/grade 0) of each specific liver histology, we observed significant inverse associations of IPA levels with lobular inflammation (*p* = 0.039) and fibrosis (*p* = 0.039) but not with steatosis (*p* = 0.985) or ballooning (*p* = 0.354) (Figure 1). When the model was adjusted for age, gender, and BMI, the association of IPA levels remained borderline significant for lobular inflammation (*p* = 0.051) and fibrosis (*p* = 0.076).

### 3.2. IPA Levels Are Markedly Reduced in Individuals with Fibrosis and without T2D

**Figure 2:**
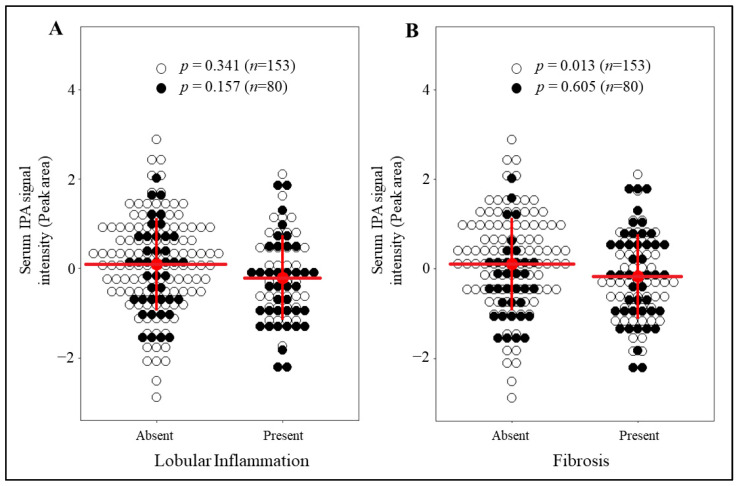
Associations of serum IPA signal intensity with lobular inflammation and fibrosis, based on presence (all except 0) or absence (stage/grade 0) of either lobular inflammation or fibrosis in individuals with or without T2D. Dot plot presents the inverse-normalized serum IPA signal intensities (*y*-axis) for (**A**) lobular inflammation and (**B**) fibrosis. The white dots are individuals without T2D, and black are those with T2D. Red lines and whiskers represent mean serum IPA signal intensity and SD respectively for whole population. General linear model (univariate) test results *p* value is shown as p for each comparison between presence or absence of each specific histological characteristic separately in those with and without T2D.

To more accurately investigate the relationship of circulating IPA and liver histology considering the already known interaction of IPA with T2D [8,13,38], a more homogeneous sub-population of individuals without T2D was selected from the current cohort (*n* = 153). In nondiabetic individuals, IPA levels were lower for those with fibrosis compared to those without fibrosis (*p* = 0.013 unadjusted model; *p* = 0.019 model adjusted for age, gender, and BMI; Figure 2A) while the presence of lobular inflammation (Figure 2B), steatosis and ballooning (Figure S2) did not associate with IPA. In addition, when the associations of IPA levels across all the stages/grades within each of the liver histology (Figure S3) were analyzed, IPA levels were found to be significantly associated with fibrosis (*p* = 0.030 unadjusted model; *p* = 0.044 model adjusted for age, gender, and BMI) for individuals without T2D. The levels of IPA were not associated with fibrosis in individuals with T2D as shown in Figure 2 and Figure S3.

### 3.3. Circulating IPA Associates with Liver Transcripts Enriched for Pathways Related to Fibrosis

**Figure 3:**
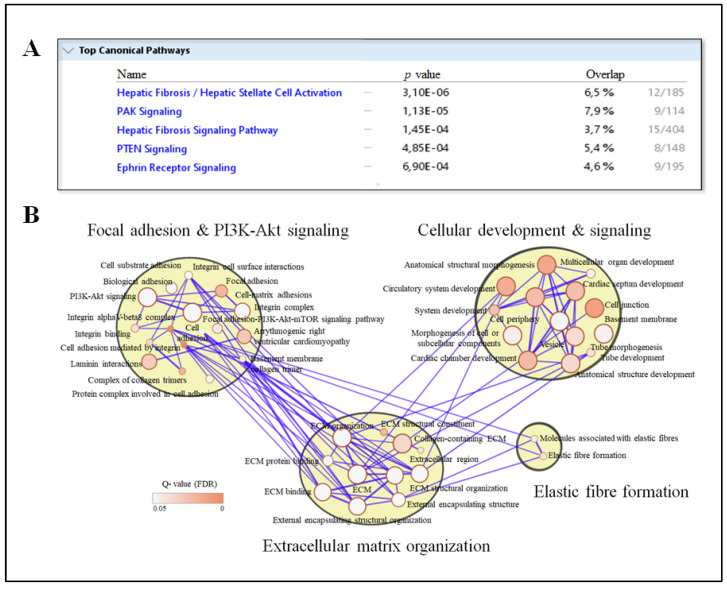
Enriched pathways corresponding to the significantly (*p* value < 0.01) associated genes with serum IPA signal intensities. (**A**) Top canonical pathways based on a total of 278 transcripts IDs and corresponding genes that were nominally associated with IPA (Ingenuity Pathway Analysis). The *p* value for the enrichment for each pathway is shown with the total overlapping genes percentage. (**B**) Enrichment map based on gene ontology and reactome terms (g:Profiler in Cytoscape). The connection between the nodes (edges) are depicted with blue lines and intensity of red color indicates the Q value (adjusted *p* value) for the enrichment score for each node.

To identify molecular mechanisms explaining the association between IPA and liver fibrosis, we investigated the associations of circulating IPA levels with the global liver transcriptomics. Analysis of the global liver gene expression identified 278 transcripts with *p* value < 0.01 that associated with the IPA levels (gene counts adjusted for technical factors, age, gender, and BMI). The top canonical pathway enriched using the Ingenuity Pathway Analysis was hepatic fibrosis/hepatic stellate cell activation (Figure 3A). In addition, g:Profiler based pathway enrichment for the corresponding transcripts revealed enrichment of the following pathways: extracellular matrix organization, focal adhesion and PI3K-Akt signaling, elastic fibers formation and cellular development and signaling (Figure 3B). The genes significantly associated with IPA levels are tabulated in Table S3. The top three genes associated (FDR *p* < 0.1) with IPA were *SLC11A1* (solute carrier family 11 member 1)*, MROH6* (maestro heat-like repeat-containing protein family member 6) and *MAPKAPK3* (mitogen-activated protein kinase-activated protein kinase 3). This also included markers of hepatic stellate cell activation, such as *ITGA3* (*p* = 0.000415).

### 3.4. IPA Reduces Cell Adhesion and Migration of LX-2 Cells

**Figure 4:**
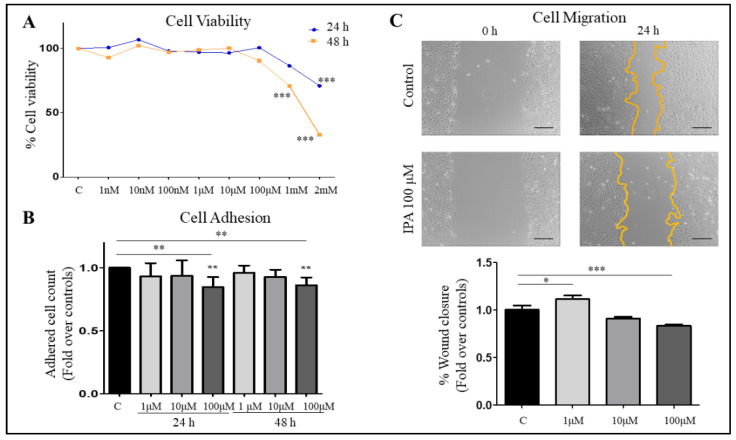
Effect of IPA on LX-2 cell viability, cell adhesion, and cell migration. (**A**) Impact on viability in LX-2 cells after IPA treatment for 24 and 48 h. Line plot represents % cell viability. (**B**) The effect of IPA on cell adhesion, bar plot presents the fold change reduction in adhered cells after IPA treatment for 24 and 48 h. (**C**) Representative images of control and IPA 100 µM at 0 and 24 h after the monolayer injury. Magnification 50× and at least 4 images for each group. Bar plot represents the % wound closure after 24 h. All the values are presented as mean ± SD, *n* = 3 independent experiments, One-way ANOVA followed by Bonferroni’s post hoc test was used for statistical comparisons. C—control, * *p* value < 0.05, ** *p* value < 0.01, *** *p* value < 0.001 compared to corresponding controls.

Next, we established a model to investigate the potential role of IPA in the regulation of stellate cells by treating LX-2 cells with varying concentrations of IPA and demonstrating that as high as 100 µM of IPA exhibits no change in cell viability after 24 and 48 h of treatment (Figure 4A). We further checked the effect of IPA on cell adhesion and cell migration, known features of HSCs activation [44], 100 μM IPA treatment significantly reduced the cell adhesion after 24 and 48 h of pre-treatment (Figure 4B). The treatment with 100 μM of IPA also significantly reduced the cell migration in LX-2 cells (Figure 4C).

### 3.5. IPA Subsides Fibrogenesis in Activated LX-2 Cells

**Figure 5:**
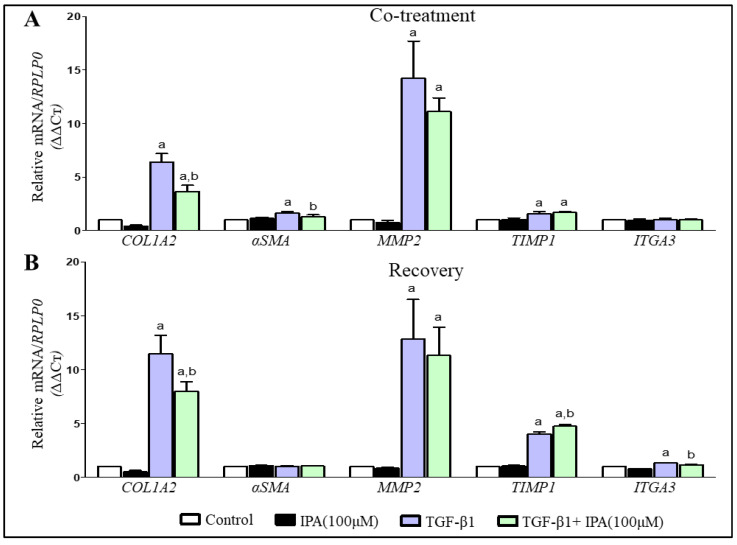
TGF-β1 induced mRNA gene expression changes and effect of IPA treatment on LX-2. Bar plot presents the relative mRNA with respect to endogenous control (*RPLP0*) after induction of LX-2 cells with TGF-β1 for 24 h, (**A**) Co-treatment with IPA for 24 h and (**B**) Recovery group, IPA treatment for 24 h after one day of cell induction with TGF-β1. All the values are presented as mean ± SD, *n* = 3 independent experiments, One-way ANOVA followed by Bonferroni’s post hoc test was used for statistical comparisons. Statistically significant results are shown with alphabets; a, *p* value < 0.05 vs. control; b, *p* value < 0.05 vs. TGF-β1.

The effect of IPA on classical markers of HSCs activation was studied using LX-2 cells with or without TGF-β1 (5 ng/mL) treatment for 24 or 48 h. In LX-2 cells treated only with IPA (100 µM), there was no change in the mRNA gene expression of classical markers of HSCs activation (Figure 5). As expected, treatment with TGF-β1 led to an increase in mRNA gene expression of *COL1A2*, *MMP2*, and *TIMP1* [45]. However, when the cells were treated with IPA (100 µM) as co-treatment with TGF-β1, we demonstrated significantly lower mRNA gene expression of *COL1A2* and *αSMA* as compared to TGF-β1 (Figure 5A). When IPA (100 µM) was added as a recovery treatment after 24 h of TGF-β1 treatment, there was a significant decrease of mRNA gene expression of *COL1A2* and *ITGA3* and an increased expression of *TIMP1* as compared to TGF-β1 (Figure 5B).

## 4. Discussion and Conclusions

In the present study, we observed that liver inflammation and fibrosis were associated with circulating levels of gut-derived metabolite IPA, especially in individuals without T2D (Figure 1 and Figure 2). Circulating levels of IPA were associated with mRNA levels of genes enriched for pathways involve in hepatic stellate cell activation and altered extracellular signaling (Figure 3), suggesting IPA can ameliorate activation of stellate cells in human liver and thus reduce fibrosis. To support this, we observed that IPA treatment is able to rescue and reduce activation of LX-2 cells stimulated by TGF-β1. Altogether, this evidence suggests that IPA is a promising candidate for further studies aimed at identifying new treatments for reversal or management of hepatic fibrogenesis.

The important clinical finding that started these analyses was the observation that liver inflammation and fibrosis, but not steatosis, were associated with circulating levels of IPA in obese individuals (Figure 1). More specifically, in obese individuals undergoing bariatric surgery, we found fasting serum IPA levels to be higher in those without fibrosis compared to those with fibrosis, a relationship that was even more evident in individuals without T2D (Figure 2). Existing evidence associate indoles and their derivatives with the improvement of gut metabolism, liver health, and liver integrity [46]. For example, circulating indole levels were found to be inversely correlated with hepatic fat content and BMI in human subjects [47]. Lower circulating IPA levels have also been found in individuals with obesity, chronic kidney diseases, T2D, atherosclerosis, and other metabolic diseases, which to an extent, is proposed to be mediated by dietary modifications affecting microbiota production [7,8,9,10,11,12,48]. Thus, our results strengthen the evidence that IPA links with development of liver diseases, as earlier reported for other metabolites such as choline, betaine and secondary bile acids [27,30,49]. Regarding the strong links between T2D and liver fibrosis [50] it is important to note that IPA levels have been associated with T2D and glucose homeostasis in rodents and humans [8,13,16,19]. Therefore, our observation that IPA associates with fibrosis in nondiabetic individuals (Figure 2) is an important finding highlighting a link between IPA and liver fibrosis independent of T2D.

Consistent with the suggestion that IPA can regulate liver fibrosis in humans, circulating IPA associated with liver transcripts enriched in hepatic stellate cell activation and altered extracellular signaling (Figure 3), which are the major players in the onset and progression of fibrosis [31,42]. Within these pathways, genes associated with fibrosis signaling, including *ITGA3*, *ITGAV*, *LAMC3*, and *COL1A2*, were negatively correlated with the circulating IPA levels. These genes belong to the family of extracellular matrix components (including integrins, laminins and collagen) and are known to be activated by steatosis derived inflammatory mediators in a fatty liver [51]. Previously, IPA has been shown to inhibit the expression of fibrogenic and collagen family genes and attenuate diet-induced NASH phenotypes in rats [22], and to induce cytostatic effect and regulate cell-cell adhesion and communication both in vivo and in vitro [52,53]. Therefore, our transcriptomic analysis of human liver strengthens the conclusion that IPA may have hepatoprotective effect in humans.

To confirm the potential of IPA to regulate activation of HSCs we demonstrated that IPA could reduce cell adhesion and migration, and partially restore mRNA expression of genes related to activation of LX-2 cells by TGF-β1 (Figure 4 and Figure 5). These responses to IPA treatment also support the role of IPA in fibrogenesis because the reduction in cell adhesion and migration could be an indicator of a more quiescent phenotype or a less activated phenotype for HSCs [33], indicating a protective effect of IPA. Liver fibrosis is a result of healing response to chronic liver injury characterized by deposition of extracellular matrix (ECM) through the activation of *COL1A2*, *αSMA* and growth factors in HSCs [31]. Thus, the reduction in the mRNA gene expression of these classical markers *COL1A2* and *aSMA* of cell activation and alterations in ECM associated genes *TIMP1* and *ITGA3* validate the ameliorative effect of IPA on HSCs. Similar changes in mRNA expression in response to IPA have been previously observed in diet-induced rodent model of NASH [22].

We have acknowledged limitations in our study. The prospective cohort cannot link our conclusions to causation. For this reason, we performed the experimental studies in LX-2 cells to obtain, at least preliminary evidence for causation. Although it is difficult to prove the clinically relevant causal relationship because of the nature of the study, in vitro validation using the cell model provides a strong suggestion that IPA can rescue hepatic fibrosis. The mechanisms of this action should be revealed in more detailed experimental studies including changes in protein levels, inflammation and functional assessment of ECM in response to IPA. Regarding the action of IPA in the liver, we agree that the availability of intrahepatic IPA content would allow us to better understand the mechanism and the metabolic outcomes at tissue-level. However, this was not possible to obtain and may also not be relevant because of the potentially low tissue concentrations. Under physiological conditions, serum IPA concentrations range from 1–10 μM in humans [19,38]. In most of the in vitro studies testing the efficacy of IPA, 100 μM and higher doses have been used [15,20,40,41]. Since we have no knowledge about the tissue concentrations in humans, we decided to use commonly used 100 μM concentration with cells. A recent study also reports contrasting acute and chronic effect of IPA on mitochondrial function in human cardiomyocytes [40] which also indicates that detailed mechanistic actions have to be investigated before proceeding to clinical studies in humans.

In conclusion, we observed that circulating levels of gut-derived metabolite IPA were associated with liver inflammation and fibrosis and with mRNA levels of genes enriched for pathways including hepatic stellate cell activation and fibrosis signaling. Studies with activated HSCs further supported the conclusion that IPA can reduce activation of stellate cells. Altogether, our findings suggests that IPA is a promising candidate for further studies aimed at identifying new treatments for reversal or management of hepatic fibrogenesis.

## Figures and Tables

**Table 1:** Clinical characteristics and liver histology of study participants according to histological liver phenotype.

	Normal Liver	Simple Steatosis	NASH	*p* ^a^
Total, *N* (men/women)	79 (20/59)	40 (9/31)	45 (18/27)	0.14
Age (years)	47.4 ± 9.7	46.5 ± 8.6	49.5 ± 9.6	0.31
BMI (kg/m^2^)	42.6 ± 5.5	43.3 ± 4.8	43.4 ± 5.4	0.61
fS-Total cholesterol (mmol/L)	4.2 ± 0.8	4.2 ± 0.9	4.5 ± 1.1	0.27
fS-LDL cholesterol (mmol/L)	2.4 ± 0.7	2.4 ± 0.9	2.5 ± 1.0	0.80
fS-HDL cholesterol (mmol/L)	1.2 ± 0.3	1.1 ± 0.2	1.2 ± 0.4	0.27
fS-Triglycerides (mmol/L)	1.3 (1.0–2.3)	1.4 (0.9–2.0)	1.6 (1.3–2.2)	0.60
fP-glucose (mmol/L)	6.0 ± 1.3	6.3 ± 1.9	7.3 ± 2.2 *	0.0005
fS-insulin (mU/L)	13.8 (7.8–18.6)	16.0 (11.0–23.4) *	20.5 (14.4–28.8) *	0.00006
Type 2 diabetes, *N* (%)	15 (18.9)	11 (27.5)	26 (57.8) *	0.00003
Lipid lowering medication, *N* (%)	22 (28.6)	9 (22.5)	20 (44.4)	0.07
Glucose lowering medication, *N* (%)	14 (17.7)	11 (25)	24 (53.3) *	0.0002
IPA levels (Inverse normalized)	0.04 ± 1.11	0.18 ± 0.93	−0.04±0.98	0.63
Steatosis grade, *N*				
<5%	79	0	0	
5–33%	0	32	13	
33–66%	0	5	19	
>66%	0	3	13	
Lobular inflammation, *N*	0	0	45	
Ballooning, *N*	0	0	37	
Fibrosis, *N* (stage range)	0	0	42 (1–3)	

Data shown as mean ± SD or median and interquartile range (IQR). fS—fasting serum, fP—fasting plasma, HDL—high-density lipoprotein, LDL—low-density lipoprotein, *N*—number of individuals, ^a^ One-way ANOVA test (continuous variable) or Chi^2^ test (categorical variable), After post hoc Bonferroni correction for multiple testing, * *p* value < 0.05 vs. normal liver.

## Supplementary Materials

The following are available online at https://www.mdpi.com/article/10.3390/nu13103509/s1, Methods S1: Reagents and chemicals, Figure S1: IPA measurement correlation, Figure S2: Associations of serum IPA signal intensity with steatosis and ballooning, based on presence (all except 0) or absence (stage/grade 0) of either steatosis or ballooning in individuals with or without T2D, Figure S3: Associations of serum IPA signal intensity across all the stages/grades within each of the liver histology with or without T2D, Table S1:Characteristics of the individuals undergoing bariatric surgery with IPA signal intensity identified using non-targeted and IPA concentrations using targeted metabolomics included in the current study, Table S2: Primer sequences, Table S3: List of liver transcripts/genes significantly associated with IPA levels.
