# Supplementary material for: Indole-3-Propionic Acid, a Gut-Derived Tryptophan Metabolite, Associates with Hepatic Fibrosis
Source: Nutrients. 2021 Oct 5;13(10):3509. doi: 10.3390/nu13103509 (PMC8538297; doi:10.3390/nu13103509)
Supplement: Supplementary file 1 [file nutrients-13-03509-s001.zip › nutrients-1374267-supplementary.pdf]

Original Article

Indole-3-propionic acid, a gut-derived tryptophan metabolite, associates with hepatic fibrosis

Ratika Sehgal[1], Mariana Ilha[1], Maija Vaittinen[1], Dorota Kaminska[1], Ville Männistö[2], Vesa Kärjä[3], Marjo Tuomainen[1], Kati Hanhineva[1,4], Stefano Romeo[5], Päivi Pajukanta[6,7], Jussi Pihlajamäki[1,8*], Vanessa D. de Mello[1*]

***shared authorship**

Methods S1. Reagents and chemicals

Dulbecco's Modified Eagle's Medium, DMEM-F12 W/L-Glutamine W/15 MM HEPES (Biowest, L0093-500), Penicillin-Streptomycin Mixture (Pen/Strep) containing 10,000 units potassium penicillin and 10,000 μg streptomycin sulfate per mL (Lonza, DE17-602E), Fetal bovine serum, FBS (Gibco, 10270-106), Dulbecco's phosphate-buffered saline, DPBS (Biowest, L0615-500), Trypsin EDTA (T4049-500), fatty acid free-Bovine serum albumin, BSA (Sigma, A8806), 3-(4,5-Dimethylthiazol-2-yl)-2,5-Diphenyltetrazolium Bromide, MTT (Sigma, M5655), Recombinant Human TGF-β1 Protein (R&D systems, 240-B-002/CF), Indole-3-propionic acid, IPA (Sigma, 220027), Dimethyl sulfoxide, DMSO (Riedel-de Haën, 34943), β-mecaptoethanol (Sigma, 63689-100ML-F), Qubit™ RNA HS Assay Kit (Thermo Fischer, Q32855), RNeasy mini RNA extraction kit (Qiagen, 74106), High Capacity cDNA Reverse Transcription Kit (Thermo Fischer, 4368813), Sensifast SYBR Lo-ROX Kit (Bioline, BIO 94050), PCR primers were purchased from Metabion, Germany).

Figure S1. IPA measurements correlation

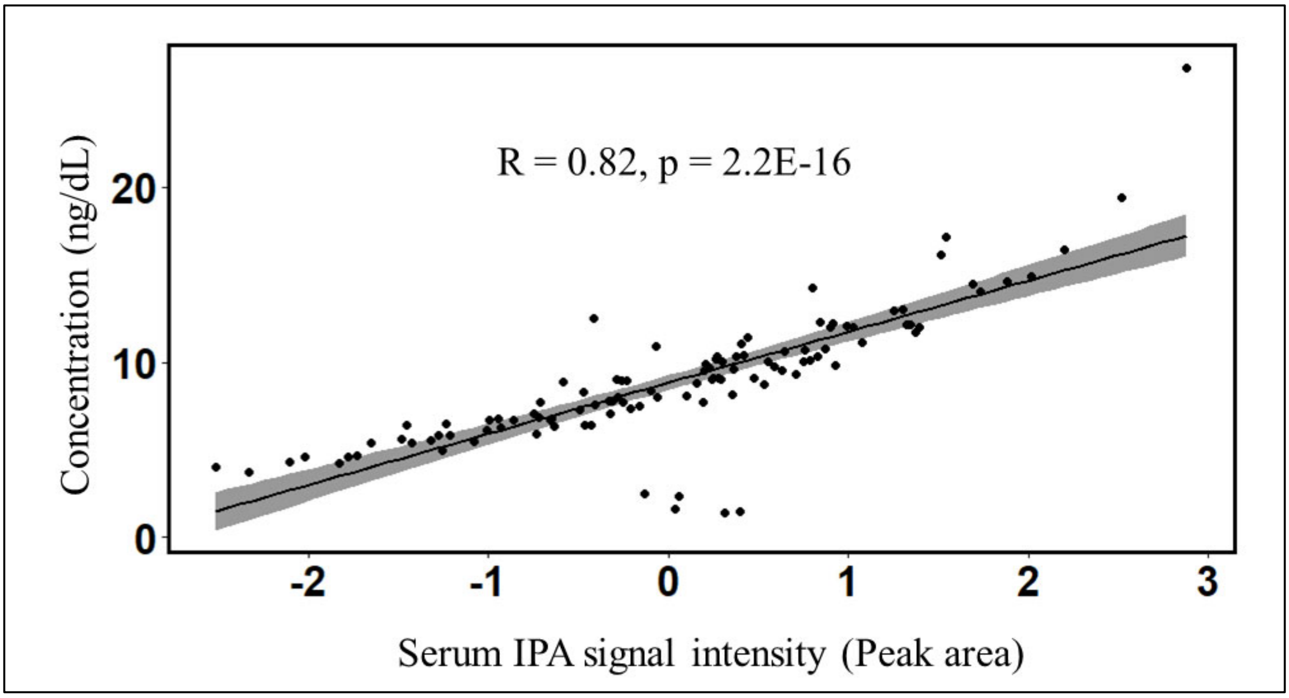

Scatter plot presents the inverse-normalized serum IPA signal intensities (x-axis) and square root-normal-IPA concentrations (ng/dL) (y-axis). Each dot represents individuals from KOBS with both the measurements.

Figure S2. Associations of serum IPA signal intensity with steatosis and ballooning, based on presence (all except 0) or absence (stage/grade 0) of either steatosis or ballooning in individuals with or without T2D

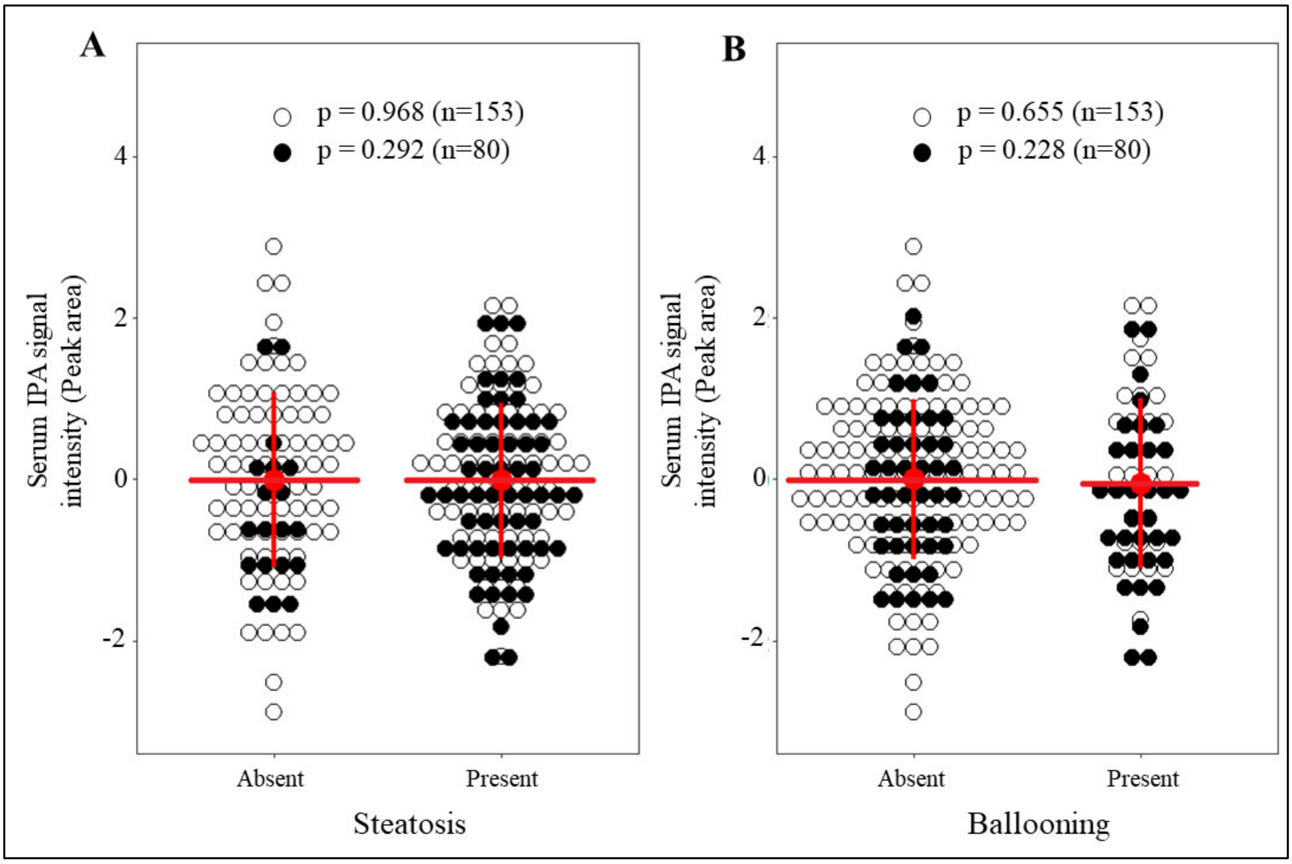

Dot plot presents the inverse-normalized serum IPA signal intensities (y-axis) for (A) Steatosis and (B) Ballooning. The white dots are individuals without T2D, and black are those with T2D. Red line and whiskers represent mean serum IPA signal intensity and SD respectively for whole population. General linear model (univariate) test results p value is shown as p for each comparison between presence or absence of each specific histological characteristic separately in those with and without T2D.

Figure S3. Associations of serum IPA signal intensity across all the stages/grades within each of the liver histology with or without T2D

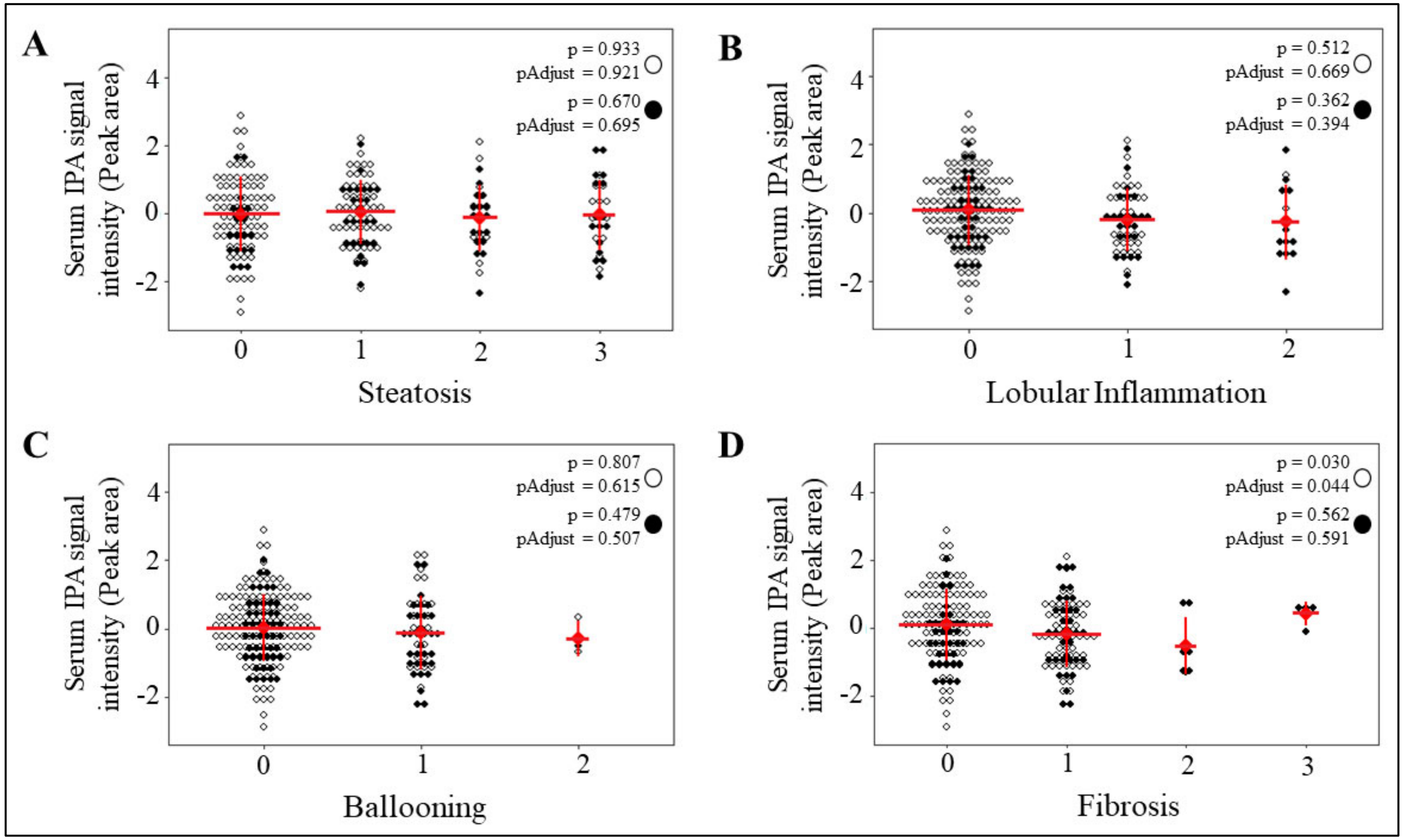

Dot plot presents the inverse-normalized serum IPA signal intensities (y-axis) across the (A) Steatosis, (B) Lobular inflammation, (C) Ballooning and (D) Fibrosis. The white dots are individuals without T2D, and black are those with T2D. Red line and whiskers represent mean serum IPA signal intensity and SD respectively for whole population. General linear model (univariate) test results are shown as p value and results for model further adjusted for age, gender, and BMI are shown as pAdjust separately in those with and without T2D.

Table S1. Characteristics of the individuals undergoing bariatric surgery with IPA signal intensity identified using non-targeted and IPA concentrations using targeted metabolomics included in the current study.

	IPA signal intensity	IPA concentration	p value
Total, N (men/women)	233 (64/169)	121 (36/85)	0.651
Age (years)	48.3 ± 9.3	48.4 ± 9.4	0.955
BMI (kg/m^2)	43.1 ± 5.4	42.9 ± 5.3	0.780
fS-Total cholesterol (mmol/l)	4.2 ± 0.9	4.2 ± 0.9	0.396
fS-LDL cholesterol (mmol/l)	2.4 ± 0.8	2.3 ± 0.8	0.480
fS-HDL cholesterol (mmol/l)	1.1 ± 0.3	1.1 ± 0.3	0.485
fS-Triglycerides (mmol/l)	1.3 (1.0 – 1.9)	1.4 (1.1 – 1.9)	0.915
fP-glucose (mmol/l)	6.5 ± 1.8	6.5 ± 1.9	0.852
fS-insulin (mU/l)	16.3 (11.0 – 23.1)	15.8 (10.5 – 24.1)	0.347
Type 2 diabetes, N (%)	80 (34.3)	40 (33.1)	0.810
Steatosis grade, N <5%	97	45	
5-33%	77	44	
33-66%	30	15	
>66%	29	17	
Lobular inflammation, N, 0	161	82	
1	56	31	
2	16	8	
Ballooning, N, 0	175	84	
1	55	37	
2	3	0	
Fibrosis, N, 0	136	63	
1	85	50	
2	8	4	

3	4	4

Data shown as mean ± SD or median (IQR). fS - fasting serum, fP - fasting plasma, HDL – High density lipoprotein, LDL – Low density lipoprotein, N – number of individuals.

Table S2. Primer sequences

Gene ID	Primer	Sequence	References
COL1A2	Forward primer	5′-TGGTCTCGGTGGGAACTTTG	
	Reverse primer	5′-CACCCTGTGGTCCAACAACT	[1]
αSMA	Forward primer	5′-AATCCTGACCCTGAAGTACC	
	Reverse primer	5′-TAGAAAGAGTGGTGCCAGAT	[1]
MMP2	Forward primer	5′-AAGTATGGCTTCTGCCCTGA	
	Reverse primer	5′-TCCATCCTGCAGTTTTCCAG	[2]
TIMP1	Forward primer	5′-CAAGATGTATAAAGGGTTCCAAGC	
	Reverse primer	5′-TCCATCCTGCAGTTTTCCAG	[2]
ITGA3	Forward primer	5′- CAGACCTGAACAATGATGGGTGG	
	Reverse primer	5′- AGCCCCTTAGAGCTACTGTGA	[3]
RPLP0	Forward primer	5′-GGCGACCTGGAAGTCCAACT	
	Reverse primer	5′-CCATCAGCACCACAGCCTTC	[4]

Table S3. List of liver transcripts/genes significantly associated with IPA levels.

Ensemble ID	Gene ID	Type	logFC	logCPM	F	P value
ENSG00000018280	*SLC11A1*	protein_coding	2.84E-06	1.119015	22.28724	4.87E-06
ENSG00000204839	*MROH6*	protein_coding	3.95E-06	2.196707	20.49504	1.12E-05
ENSG00000114738	*MAPKAPK3*	protein_coding	1.57E-06	3.656099	19.3433	1.91E-05
ENSG00000025770	*NCAPH2*	protein_coding	1.08E-06	3.843419	18.54083	2.8E-05
ENSG00000284879	*AC133644.3*	processed_transcript	2.36E-06	1.110261	17.09499	5.56E-05
ENSG00000148541	*FAM13C*	protein_coding	-2.2E-06	1.671719	14.80407	0.000168
ENSG00000249970	*AC074250.1*	processed_pseudogene	2.54E-06	1.655974	14.6788	0.000179
ENSG00000154553	*PDLIM3*	protein_coding	-3.5E-06	1.22309	14.67117	0.000179
ENSG00000177058	*SLC38A9*	protein_coding	-8E-07	3.953832	14.67047	0.000179
ENSG00000211455	*STK38L*	protein_coding	-8.7E-07	4.0143	13.92165	0.000259
ENSG00000103449	*SALL1*	protein_coding	1.57E-06	5.23946	13.27405	0.000357
ENSG00000115380	*EFEMP1*	protein_coding	-4E-06	2.386421	13.14355	0.00038

ENSG00000114737	*CISH*	protein_coding	4.46E-06	3.815758	13.13453	0.000382
ENSG00000087589	*CASS4*	protein_coding	1.53E-06	0.928277	13.08869	0.000391
ENSG00000005884	*ITGA3*	protein_coding	-2.1E-06	2.679976	12.96834	0.000415
ENSG00000139329	*LUM*	protein_coding	-2.9E-06	4.327572	12.41848	0.000545
ENSG00000121989	*ACVR2A*	protein_coding	-7.1E-07	4.207775	12.30649	0.000577
ENSG00000095794	*CREM*	protein_coding	1.11E-06	3.414095	12.25886	0.000591
ENSG00000248050	*AC079061.1*	antisense	7.26E-06	2.579942	12.22562	0.000601
ENSG00000249173	*LINC01093*	lincRNA	2.34E-06	5.261332	12.22021	0.000602
ENSG00000260244	*AC104083.1*	sense_overlapping	-1.8E-06	2.814562	12.15701	0.000622
ENSG00000174807	*CD248*	protein_coding	-2.7E-06	0.286128	12.11757	0.000634
ENSG00000112655	*PTK7*	protein_coding	-2.3E-06	1.357102	12.09881	0.00064
ENSG00000119535	*CSF3R*	protein_coding	2.23E-06	3.208674	12.03747	0.00066
ENSG00000103319	*EEF2K*	protein_coding	8.22E-07	4.81295	11.93712	0.000694
ENSG00000246985	*SOCS2-AS1*	processed_transcript	3.55E-06	0.732009	11.9071	0.000704
ENSG00000170085	*SIMC1*	protein_coding	1.4E-06	2.985512	11.7922	0.000746
ENSG00000131711	*MAP1B*	protein_coding	-2.3E-06	3.284154	11.78913	0.000747
ENSG00000172348	*RCAN2*	protein_coding	-2.3E-06	2.174652	11.74802	0.000763
ENSG00000044524	*EPHA3*	protein_coding	-2.3E-06	3.952663	11.69574	0.000783
ENSG00000267395	*DM1-AS*	antisense	1.76E-06	0.639942	11.47357	0.000876
ENSG00000274605	*AL355338.1*	lincRNA	1.69E-06	2.181794	11.36476	0.000925
ENSG00000076716	*GPC4*	protein_coding	-2.7E-06	1.153597	11.25681	0.000977
ENSG00000118298	*CA14*	protein_coding	3.23E-06	1.12918	11.10947	0.001053
ENSG00000225335	*AC016027.1*	antisense	2.05E-06	0.323028	10.98718	0.00112
ENSG00000184347	*SLIT3*	protein_coding	-2.8E-06	2.961303	10.90849	0.001166
ENSG00000152359	*POC5*	protein_coding	-1E-06	2.227086	10.87867	0.001183
ENSG00000163220	*S100A9*	protein_coding	3.73E-06	2.236294	10.82163	0.001218
ENSG00000112144	*ICK*	protein_coding	-5.9E-07	4.15351	10.81921	0.00122
ENSG00000082269	*FAM135A*	protein_coding	-8E-07	3.566426	10.80434	0.001229
ENSG00000147257	*GPC3*	protein_coding	-5.2E-06	1.276956	10.79281	0.001236
ENSG00000205959	*AC105345.1*	lincRNA	1.84E-06	0.714072	10.75383	0.001261
ENSG00000153956	*CACNA2D1*	protein_coding	-1.9E-06	2.256197	10.71389	0.001287
ENSG00000105327	*BBC3*	protein_coding	-2.5E-06	0.552811	10.69901	0.001296
ENSG00000243466	*IGKV1-5*	IG_V_gene	-3.7E-06	2.355315	10.68921	0.001303
ENSG00000118620	*ZNF430*	protein_coding	-1.5E-06	2.024415	10.64507	0.001332

ENSG00000067798	*NAV3*	protein_coding	-2.7E-06	2.691602	10.62453	0.001346
ENSG00000121440	*PDZRN3*	protein_coding	-2E-06	1.629408	10.61293	0.001354
ENSG00000114735	*HEMK1*	protein_coding	9.85E-07	5.441855	10.59032	0.00137
ENSG00000188536	*HBA2*	protein_coding	4.78E-06	3.535438	10.54219	0.001404
ENSG00000145147	*SLIT2*	protein_coding	-1.9E-06	2.387672	10.53688	0.001408
ENSG00000137269	*LRRC1*	protein_coding	-2.6E-06	0.368831	10.35686	0.001543
ENSG00000106624	*AEBP1*	protein_coding	-2.8E-06	4.822716	10.34966	0.001549
ENSG00000164659	*KIAA1324L*	protein_coding	-2.2E-06	1.453986	10.34103	0.001556
ENSG00000122557	*HERPUD2*	protein_coding	-6.8E-07	4.261798	10.32076	0.001572
ENSG00000123096	*SSPN*	protein_coding	-2.8E-06	1.305391	10.28643	0.0016
ENSG00000163959	*SLC51A*	protein_coding	1.82E-06	4.902774	10.20047	0.001672
ENSG00000280344	*AC009166.2*	TEC	1.66E-06	1.763798	10.19525	0.001676
ENSG00000284779	*IGF2*	protein_coding	1.05E-06	3.550311	10.12326	0.001739
ENSG00000164087	*POC1A*	protein_coding	1.39E-06	0.950629	10.11646	0.001745
ENSG00000200959	*SNORA74A*	snoRNA	-2.7E-06	3.491249	10.08201	0.001776
ENSG00000102349	*KLF8*	protein_coding	-1.8E-06	1.836285	10.05928	0.001797
ENSG00000115523	*GNLY*	protein_coding	3E-06	2.215801	10.05662	0.001799
ENSG00000137831	*UACA*	protein_coding	-8.4E-07	5.272735	10.0366	0.001818
ENSG00000200156	*RNU5B-1*	snRNA	-3E-06	2.897557	10.0148	0.001838
ENSG00000153214	*TMEM87B*	protein_coding	-8.3E-07	3.663641	10.01421	0.001839
ENSG00000050165	*DKK3*	protein_coding	-2.5E-06	2.50636	10.0013	0.001851
ENSG00000078902	*TOLLIP*	protein_coding	7.06E-07	5.902184	9.976223	0.001875
ENSG00000134531	*EMP1*	protein_coding	-2.9E-06	2.108945	9.944759	0.001906
ENSG00000143641	*GALNT2*	protein_coding	1.25E-06	6.379881	9.802961	0.00205
ENSG00000183722	*LHFPL6*	protein_coding	-2E-06	3.017355	9.752516	0.002103
ENSG00000144857	*BOC*	protein_coding	-3.1E-06	1.150561	9.646307	0.002222
ENSG00000163531	*NFASC*	protein_coding	-2.1E-06	4.533246	9.634301	0.002235
ENSG00000188158	*NHS*	protein_coding	-1.7E-06	0.642654	9.611587	0.002262
ENSG00000150681	*RGS18*	protein_coding	2.34E-06	1.16377	9.61129	0.002262
ENSG00000182836	*PLCXD3*	protein_coding	-2.4E-06	2.51345	9.590739	0.002286
ENSG00000101384	*JAG1*	protein_coding	-1.7E-06	4.36437	9.587432	0.00229
ENSG00000198542	*ITGBL1*	protein_coding	-3.4E-06	2.534599	9.580099	0.002299
ENSG00000119720	*NRDE2*	protein_coding	8.99E-07	5.131679	9.5673	0.002314
ENSG00000274750	*HIST1H3E*	protein_coding	-1.5E-06	1.148496	9.552954	0.002331

ENSG00000169604	*ANTXR1*	protein_coding	-2E-06	3.682345	9.509745	0.002384
ENSG00000137077	*CCL21*	protein_coding	-2.8E-06	2.339562	9.449208	0.002459
ENSG00000085365	*SCAMP1*	protein_coding	-9E-07	4.032782	9.448159	0.002461
ENSG00000154096	*THY1*	protein_coding	-3.3E-06	1.576305	9.425157	0.00249
ENSG00000140545	*MFGE8*	protein_coding	-2E-06	2.569044	9.35078	0.002587
ENSG00000166548	*TK2*	protein_coding	6.33E-07	4.973501	9.345472	0.002595
ENSG00000115904	*SOS1*	protein_coding	-4.5E-07	5.483013	9.27655	0.002689
ENSG00000050555	*LAMC3*	protein_coding	-2.6E-06	3.271781	9.273244	0.002693
ENSG00000143603	*KCNN3*	protein_coding	-2.3E-06	1.286506	9.272663	0.002694
ENSG00000058272	*PPP1R12A*	protein_coding	-5.9E-07	5.229586	9.264378	0.002706
ENSG00000171747	*LGALS4*	protein_coding	-3.7E-06	4.994463	9.238331	0.002742
ENSG00000084636	*COL16A1*	protein_coding	-2.6E-06	3.117652	9.203396	0.002793
ENSG00000167910	*CYP7A1*	protein_coding	-5.1E-06	5.501106	9.193159	0.002807
ENSG00000172465	*TCEAL1*	protein_coding	-1.1E-06	1.874574	9.183305	0.002822
ENSG00000107317	*PTGDS*	protein_coding	-3.4E-06	1.458723	9.138879	0.002887
ENSG00000113248	*PCDHB15*	protein_coding	-1.7E-06	0.503104	9.11151	0.002929
ENSG00000185630	*PBX1*	protein_coding	-1.6E-06	5.002058	9.107266	0.002935
ENSG00000152413	*HOMER1*	protein_coding	-2.2E-06	0.559963	9.097398	0.00295
ENSG00000103148	*NPRL3*	protein_coding	8.97E-07	3.926371	9.093916	0.002956
ENSG00000116194	*ANGPTL1*	protein_coding	-1.8E-06	3.859418	9.078949	0.002979
ENSG00000113721	*PDGFRB*	protein_coding	-1.4E-06	5.278279	9.072411	0.002989
ENSG00000166963	*MAP1A*	protein_coding	-2.5E-06	0.52824	9.071829	0.00299
ENSG00000263884	*AP000845.1*	lincRNA	1.49E-06	0.209356	9.066536	0.002998
ENSG00000196569	*LAMA2*	protein_coding	-1.7E-06	4.840701	9.052215	0.00302
ENSG00000068650	*ATP11A*	protein_coding	7.46E-07	5.627247	8.962975	0.003164
ENSG00000210100	*MT-TI*	Mt_tRNA	-3.2E-06	1.999111	8.942618	0.003197
ENSG00000182359	*KBTBD3*	protein_coding	-1.1E-06	1.896776	8.930177	0.003218
ENSG00000156535	*CD109*	protein_coding	-2.1E-06	2.293826	8.929866	0.003218
ENSG00000163590	*PPM1L*	protein_coding	-1.3E-06	4.044627	8.896158	0.003275
ENSG00000240891	*PLCXD2*	protein_coding	9.4E-07	2.968763	8.883325	0.003297
ENSG00000163520	*FBLN2*	protein_coding	-2E-06	2.544001	8.881845	0.0033
ENSG00000157637	*SLC38A10*	protein_coding	6.95E-07	6.64892	8.869279	0.003322
ENSG00000213020	*ZNF611*	protein_coding	-9.6E-07	2.929945	8.851218	0.003353
ENSG00000018625	*ATP1A2*	protein_coding	-2.9E-06	1.280614	8.811509	0.003423

ENSG00000189195	*BTBD8*	protein_coding	-9.8E-07	2.96701	8.789286	0.003463
ENSG00000071967	*CYBRD1*	protein_coding	-1.7E-06	4.647761	8.786626	0.003468
ENSG00000072952	*MRVI1*	protein_coding	-2.1E-06	2.147302	8.723845	0.003583
ENSG00000150636	*CCDC102B*	protein_coding	-1.6E-06	2.038658	8.646751	0.00373
ENSG00000151067	*CACNA1C*	protein_coding	-2.4E-06	2.528584	8.629304	0.003764
ENSG00000175137	*SH3BP5L*	protein_coding	7.21E-07	3.911877	8.629284	0.003764
ENSG00000130300	*PLVAP*	protein_coding	-2E-06	1.834851	8.626891	0.003769
ENSG00000137809	*ITGA11*	protein_coding	-1.4E-06	1.936862	8.582336	0.003858
ENSG00000196172	*ZNF681*	protein_coding	-1.9E-06	0.379179	8.559463	0.003904
ENSG00000144810	*COL8A1*	protein_coding	-2.6E-06	0.817207	8.537921	0.003948
ENSG00000186479	*RGS7BP*	protein_coding	-2.2E-06	1.386338	8.530713	0.003963
ENSG00000033170	*FUT8*	protein_coding	-1.1E-06	2.662673	8.520891	0.003984
ENSG00000143546	*S100A8*	protein_coding	3.64E-06	0.936443	8.512234	0.004002
ENSG00000154065	*ANKRD29*	protein_coding	-2.3E-06	1.347326	8.505798	0.004015
ENSG00000233836	*AC139769.1*	transcribed_unprocessed_pseudogene	-2.5E-06	0.699237	8.501805	0.004024
ENSG00000076706	*MCAM*	protein_coding	-2E-06	3.224506	8.493396	0.004041
ENSG00000160075	*SSU72*	protein_coding	4.56E-07	5.081634	8.485667	0.004058
ENSG00000099139	*PCSK5*	protein_coding	-1.9E-06	2.780303	8.466437	0.004099
ENSG00000113657	*DPYSL3*	protein_coding	-2.1E-06	2.83635	8.45541	0.004122
ENSG00000214264	*KCTD9P4*	processed_pseudogene	2.54E-06	2.607101	8.450629	0.004133
ENSG00000205726	*ITSN1*	protein_coding	6.82E-07	5.784821	8.436858	0.004163
ENSG00000107362	*ABHD17B*	protein_coding	-6.6E-07	3.410442	8.435935	0.004165
ENSG00000147852	*VLDLR*	protein_coding	-2.6E-06	1.318024	8.42989	0.004178
ENSG00000164342	*TLR3*	protein_coding	-1.1E-06	3.664322	8.419753	0.0042
ENSG00000188290	*HES4*	protein_coding	-2E-06	0.2896	8.414237	0.004212
ENSG00000137819	*PAQR5*	protein_coding	-2.2E-06	1.210912	8.401254	0.004241
ENSG00000133392	*MYH11*	protein_coding	-3E-06	4.608934	8.361327	0.004331
ENSG00000171150	*SOCS5*	protein_coding	-5.5E-07	3.882494	8.35799	0.004338
ENSG00000138172	*CALHM2*	protein_coding	-1.6E-06	1.530233	8.356226	0.004342
ENSG00000123352	*SPATS2*	protein_coding	-9.7E-07	2.588652	8.354516	0.004346
ENSG00000113971	*NPHP3*	protein_coding	-9.9E-07	3.504577	8.340747	0.004378
ENSG00000269825	*AC022150.4*	sense_intronic	-1.9E-06	0.987782	8.329791	0.004403
ENSG00000150630	*VEGFC*	protein_coding	-1.7E-06	1.241819	8.306073	0.004458
ENSG00000143196	*DPT*	protein_coding	-2.8E-06	2.411336	8.242784	0.004609

ENSG00000102287	*GABRE*	protein_coding	-2.3E-06	3.266803	8.238715	0.004619
ENSG00000198521	*ZNF43*	protein_coding	-1.3E-06	2.537952	8.230705	0.004638
ENSG00000108691	*CCL2*	protein_coding	-2.5E-06	0.906991	8.184086	0.004753
ENSG00000115942	*ORC2*	protein_coding	1.02E-06	4.403352	8.180559	0.004762
ENSG00000138448	*ITGAV*	protein_coding	-1E-06	5.547642	8.175468	0.004775
ENSG00000206077	*ZDHHC11B*	protein_coding	2.85E-06	2.955973	8.148894	0.004842
ENSG00000232442	*MHENCR*	antisense	1.25E-06	0.905764	8.103784	0.004958
ENSG00000211660	*IGLV2-23*	IG_V_gene	-3E-06	1.61047	8.079737	0.005021
ENSG00000109339	*MAPK10*	protein_coding	-1.5E-06	3.277441	8.062237	0.005068
ENSG00000188818	*ZDHHC11*	protein_coding	2.39E-06	2.72473	8.055938	0.005085
ENSG00000088356	*PDRG1*	protein_coding	8.35E-07	1.703354	7.99843	0.005241
ENSG00000138621	*PPCDC*	protein_coding	8.04E-07	2.868271	7.991743	0.00526
ENSG00000140365	*COMMD4*	protein_coding	5.95E-07	3.57823	7.989935	0.005265
ENSG00000271347	*AC124312.5*	sense_intronic	-1.3E-06	1.105468	7.987788	0.005271
ENSG00000151458	*ANKRD50*	protein_coding	-8.5E-07	4.714744	7.972044	0.005314
ENSG00000112936	*C7*	protein_coding	-2.5E-06	7.379265	7.918834	0.005466
ENSG00000143889	*HNRNPLL*	protein_coding	-6.1E-07	3.695477	7.889142	0.005552
ENSG00000068366	*ACSL4*	protein_coding	-3.9E-06	4.560876	7.884741	0.005565
ENSG00000213903	*LTB4R*	protein_coding	1.05E-06	3.139378	7.862652	0.00563
ENSG00000118785	*SPP1*	protein_coding	-3.8E-06	2.913241	7.860635	0.005636
ENSG00000150907	*FOXO1*	protein_coding	1.43E-06	5.312771	7.853529	0.005658
ENSG00000188153	*COL4A5*	protein_coding	-1.6E-06	3.161108	7.848165	0.005674
ENSG00000206538	*VGLL3*	protein_coding	-3E-06	1.181391	7.834179	0.005716
ENSG00000143702	*CEP170*	protein_coding	-6.3E-07	4.195057	7.830027	0.005728
ENSG00000211668	*IGLV2-11*	IG_V_gene	-3.4E-06	1.179822	7.829836	0.005729
ENSG00000128567	*PODXL*	protein_coding	-1.9E-06	3.830004	7.824736	0.005744
ENSG00000095015	*MAP3K1*	protein_coding	-6.8E-07	4.85003	7.809535	0.005791
ENSG00000153558	*FBXL2*	protein_coding	-2.2E-06	0.167565	7.808133	0.005795
ENSG00000159307	*SCUBE1*	protein_coding	-4.1E-06	2.250161	7.789291	0.005853
ENSG00000158258	*CLSTN2*	protein_coding	-2.5E-06	2.651547	7.763948	0.005932
ENSG00000125257	*ABCC4*	protein_coding	-1.9E-06	2.665024	7.751617	0.005971
ENSG00000169435	*RASSF6*	protein_coding	-2.9E-06	0.773818	7.74533	0.005991
ENSG00000081760	*AACS*	protein_coding	-1.5E-06	2.527871	7.709513	0.006106
ENSG00000163322	*ABRAXAS1*	protein_coding	-1E-06	2.269126	7.705015	0.00612

ENSG00000170624	*SGCD*	protein_coding	-2E-06	2.48755	7.687245	0.006178
ENSG00000198182	*ZNF607*	protein_coding	-1.5E-06	1.026321	7.667142	0.006244
ENSG00000276791	*AC092117.1*	lincRNA	1.1E-06	1.650386	7.665881	0.006248
ENSG00000115252	*PDE1A*	protein_coding	-1.4E-06	2.396499	7.64427	0.00632
ENSG00000196705	*ZNF431*	protein_coding	-1.2E-06	2.917395	7.639087	0.006338
ENSG00000077713	*SLC25A43*	protein_coding	-8.9E-07	2.780341	7.628036	0.006375
ENSG00000273148	*AL035563.1*	lincRNA	1.21E-06	0.245218	7.625977	0.006382
ENSG00000166165	*CKB*	protein_coding	-1.8E-06	1.053394	7.570211	0.006574
ENSG00000137962	*ARHGAP29*	protein_coding	-7.9E-07	6.365542	7.56096	0.006606
ENSG00000164609	*SLU7*	protein_coding	-6.3E-07	4.064668	7.542275	0.006672
ENSG00000091986	*CCDC80*	protein_coding	-2.8E-06	3.008681	7.53943	0.006682
ENSG00000105855	*ITGB8*	protein_coding	-2.1E-06	2.362634	7.536668	0.006692
ENSG00000101290	*CDS2*	protein_coding	5.95E-07	5.74685	7.523654	0.006738
ENSG00000181472	*ZBTB2*	protein_coding	-8.3E-07	2.416747	7.504859	0.006806
ENSG00000152291	*TGOLN2*	protein_coding	7.26E-07	7.592884	7.503241	0.006812
ENSG00000135919	*SERPINE2*	protein_coding	-1.6E-06	2.462149	7.499313	0.006826
ENSG00000063854	*HAGH*	protein_coding	6.88E-07	6.514672	7.495629	0.006839
ENSG00000231047	*GCNT1P3*	processed_pseudogene	2.57E-06	1.834057	7.457447	0.00698
ENSG00000147642	*SYBU*	protein_coding	2.61E-06	4.940976	7.451657	0.007001
ENSG00000164219	*PGGT1B*	protein_coding	-6E-07	4.724315	7.451022	0.007003
ENSG00000128606	*LRRC17*	protein_coding	-1.5E-06	1.295016	7.450621	0.007005
ENSG00000161298	*ZNF382*	protein_coding	-1.6E-06	1.334508	7.438505	0.00705
ENSG00000063978	*RNF4*	protein_coding	4.39E-07	4.215912	7.431859	0.007075
ENSG00000188981	*MSANTD1*	protein_coding	1.24E-06	1.160087	7.418162	0.007127
ENSG00000172845	*SP3*	protein_coding	-5E-07	5.947948	7.40904	0.007162
ENSG00000170017	*ALCAM*	protein_coding	-7.8E-07	6.229886	7.382192	0.007265
ENSG00000164300	*SERINC5*	protein_coding	7.82E-07	7.278581	7.37111	0.007308
ENSG00000182050	*MGAT4C*	protein_coding	-3E-06	0.958921	7.364819	0.007332
ENSG00000176658	*MYO1D*	protein_coding	-6.7E-07	4.90959	7.361353	0.007346
ENSG00000087074	*PPP1R15A*	protein_coding	-1.1E-06	2.747745	7.360224	0.00735
ENSG00000267444	*SMUG1P1*	processed_pseudogene	1.64E-06	2.508481	7.347263	0.007401
ENSG00000223960	*AC009948.1*	antisense	-1.2E-06	1.171593	7.333931	0.007454
ENSG00000283526	*PRRT1B*	protein_coding	1.53E-06	1.27759	7.288779	0.007636
ENSG00000285854	*AC010197.2*	protein_coding	1.46E-06	1.751771	7.284321	0.007654

ENSG00000164338	*UTP15*	protein_coding	-7.7E-07	2.801982	7.272226	0.007703
ENSG00000141384	*TAF4B*	protein_coding	-1.7E-06	0.23516	7.266011	0.007729
ENSG00000188483	*IER5L*	protein_coding	-2.2E-06	0.805797	7.261108	0.007749
ENSG00000156603	*MED19*	protein_coding	-9.7E-07	1.360327	7.255665	0.007772
ENSG00000173546	*CSPG4*	protein_coding	-2.3E-06	2.218238	7.244811	0.007817
ENSG00000204291	*COL15A1*	protein_coding	-2.9E-06	1.138766	7.23644	0.007852
ENSG00000101333	*PLCB4*	protein_coding	-2.3E-06	0.686582	7.234415	0.00786
ENSG00000169018	*FEM1B*	protein_coding	-5E-07	5.440382	7.231402	0.007873
ENSG00000150787	*PTS*	protein_coding	1.24E-06	4.153229	7.228245	0.007886
ENSG00000109929	*SC5D*	protein_coding	-1.8E-06	6.8491	7.220879	0.007917
ENSG00000252947	*SCARNA1*	scaRNA	-2.3E-06	0.57656	7.193561	0.008034
ENSG00000267838	*AC245884.8*	lincRNA	1.29E-06	0.780893	7.192755	0.008037
ENSG00000140092	*FBLN5*	protein_coding	-2.1E-06	3.90296	7.185245	0.00807
ENSG00000153094	*BCL2L11*	protein_coding	1.34E-06	4.229844	7.184114	0.008075
ENSG00000119681	*LTBP2*	protein_coding	-2.3E-06	3.396975	7.179832	0.008093
ENSG00000100767	*PAPLN*	protein_coding	-2.1E-06	2.73615	7.179759	0.008093
ENSG00000153246	*PLA2R1*	protein_coding	-1.3E-06	3.469199	7.178906	0.008097
ENSG00000101132	*PFDN4*	protein_coding	-1.3E-06	1.737487	7.17059	0.008133
ENSG00000132122	*SPATA6*	protein_coding	-1.1E-06	2.48557	7.161409	0.008173
ENSG00000140937	*CDH11*	protein_coding	-2.3E-06	1.737493	7.156423	0.008195
ENSG00000213096	*ZNF254*	protein_coding	-8.1E-07	4.525502	7.124629	0.008335
ENSG00000112782	*CLIC5*	protein_coding	-2.3E-06	0.731432	7.10183	0.008438
ENSG00000141505	*ASGR1*	protein_coding	5.6E-07	7.939537	7.097394	0.008458
ENSG00000135372	*NAT10*	protein_coding	4.75E-07	4.177947	7.080114	0.008536
ENSG00000114270	*COL7A1*	protein_coding	9.78E-06	5.419507	7.079912	0.008537
ENSG00000133606	*MKRN1*	protein_coding	3.7E-07	4.956048	7.071247	0.008577
ENSG00000198155	*ZNF876P*	transcribed_unprocessed_pseudogene	-1.2E-06	0.618828	7.070757	0.008579
ENSG00000244734	*HBB*	protein_coding	3.92E-06	5.916706	7.061661	0.008621
ENSG00000202538	*RNU4-2*	snRNA	-2.1E-06	7.681978	7.054696	0.008653
ENSG00000124279	*FASTKD3*	protein_coding	-8E-07	2.246395	7.054479	0.008654
ENSG00000145362	*ANK2*	protein_coding	-1.5E-06	2.659633	7.049814	0.008676
ENSG00000198324	*PHETA1*	protein_coding	1.08E-06	2.734306	7.030372	0.008767
ENSG00000134317	*GRHL1*	protein_coding	1.42E-06	3.993837	7.018095	0.008825
ENSG00000010319	*SEMA3G*	protein_coding	-2.4E-06	1.578617	7.013924	0.008845

ENSG00000165617	*DACT1*	protein_coding	-1.6E-06	1.616348	7.003704	0.008893
ENSG00000230630	*DNM3OS*	antisense	-1.6E-06	2.345395	6.98959	0.008961
ENSG00000162613	*FUBP1*	protein_coding	-4.6E-07	5.455007	6.98702	0.008973
ENSG00000079102	*RUNX1T1*	protein_coding	-1.4E-06	2.799719	6.96684	0.009071
ENSG00000165355	*FBXO33*	protein_coding	-6.8E-07	2.875549	6.965313	0.009078
ENSG00000143995	*MEIS1*	protein_coding	-8.5E-07	3.481437	6.959375	0.009107
ENSG00000176853	*FAM91A1*	protein_coding	-4.6E-07	5.606559	6.958268	0.009113
ENSG00000280306	*AC137056.2*	TEC	1.73E-06	4.06361	6.958252	0.009113
ENSG00000134962	*KLB*	protein_coding	-1.7E-06	5.155689	6.957053	0.009119
ENSG00000166024	*R3HCC1L*	protein_coding	-4.4E-07	3.650162	6.953241	0.009137
ENSG00000109265	*KIAA1211*	protein_coding	-2.1E-06	0.806263	6.929287	0.009255
ENSG00000163354	*DCST2*	protein_coding	1.45E-06	0.715982	6.909442	0.009355
ENSG00000163431	*LMOD1*	protein_coding	-2.7E-06	1.23824	6.904573	0.009379
ENSG00000197461	*PDGFA*	protein_coding	-1.8E-06	1.329107	6.897921	0.009413
ENSG00000182963	*GJC1*	protein_coding	-1.9E-06	1.159784	6.897905	0.009413
ENSG00000189420	*ZFP92*	protein_coding	-1.4E-06	0.795514	6.891818	0.009444
ENSG00000168743	*NPNT*	protein_coding	-2.3E-06	2.244755	6.890697	0.009449
ENSG00000152580	*IGSF10*	protein_coding	-2.5E-06	2.055844	6.871951	0.009545
ENSG00000102452	*NALCN*	protein_coding	-2.6E-06	1.318044	6.869628	0.009557
ENSG00000124587	*PEX6*	protein_coding	8.56E-07	4.578993	6.866927	0.009571
ENSG00000135930	*EIF4E2*	protein_coding	4.65E-07	4.734269	6.859873	0.009607
ENSG00000067715	*SYT1*	protein_coding	1.4E-06	1.015187	6.856321	0.009626
ENSG00000143013	*LMO4*	protein_coding	-6E-07	3.901808	6.837223	0.009725
ENSG00000154134	*ROBO3*	protein_coding	-1.6E-06	1.218562	6.819896	0.009816
ENSG00000136237	*RAPGEF5*	protein_coding	-9.6E-07	5.581262	6.819154	0.00982
ENSG00000139910	*NOVA1*	protein_coding	-1.8E-06	0.539668	6.810873	0.009864
ENSG00000176903	*PNMA1*	protein_coding	-1.2E-06	1.457738	6.806187	0.009889
ENSG00000204386	*NEU1*	protein_coding	-7.3E-07	3.992637	6.788035	0.009986

logFC – log Fold change, logCPM – log counts per million, F – F statistics.

References

[1] Miranda, J.F.; Scarinci, L.D.; Ramos, L.F.; Silva, C.M.; Gonçalves, L.R.; de Morais, P.F.; Malaspina, O.; Moraes, K.C.M. The Modulatory Effect of Triclosan on the Reversion of the Activated Phenotype of LX-2 Hepatic Stellate Cells. J. Biochem. Mol. Toxicol. **2020**, *34*, e22413.

[2] Robert, S.; Gicquel, T.; Bodin, A.; Lagente, V.; Boichot, E. Characterization of the MMP/TIMP Imbalance and Collagen Production Induced by IL-1β Or TNF-A Release from Human Hepatic Stellate Cells. PLOS ONE **2016**, *11*, e0153118.

[3] He, X.; Tao, H.; Hu, Z.; Ma, Y.; Xu, J.; Wang, H.; Xia, Y.; Li, L.; Fei, B.; Li, Y. *et al.* Expression of Galectin-1 in Carcinoma-Associated Fibroblasts Promotes Gastric Cancer Cell Invasion through Upregulation of Integrin B1. Cancer science **2014**, *105*, 1402-1410.

[4] Kaminska, D.; Hämäläinen, M.; Cederberg, H.; Käkelä, P.; Venesmaa, S.; Miettinen, P.; Ilves, I.; Herzig, K.; Kolehmainen, M.; Karhunen, L. *et al.* Adipose Tissue INSR Splicing in Humans Associates with Fasting Insulin Level and is Regulated by Weight Loss. Diabetologia **2014**, *57*, 347-351.
